# Cross-Linking Agents in Three-Component Materials Dedicated to Biomedical Applications: A Review

**DOI:** 10.3390/polym16182679

**Published:** 2024-09-23

**Authors:** Sylwia Grabska-Zielińska

**Affiliations:** Faculty of Chemical Technology and Engineering, Bydgoszcz University of Science and Technology, Seminaryjna 3, 85-326 Bydgoszcz, Poland; sylwia.grabska-zielinska@pbs.edu.pl

**Keywords:** biopolymers, cross-linking, modification, modified materials, ternary materials

## Abstract

In biomaterials research, using one or two components to prepare materials is common. However, there is a growing interest in developing materials composed of three components, as these can offer enhanced physicochemical properties compared to those consisting of one or two components. The introduction of a third component can significantly improve the mechanical strength, biocompatibility, and functionality of the resulting materials. Cross-linking is often employed to further enhance these properties, with chemical cross-linking agents being the most widely used method. This article provides an overview of the chemical agents utilized in the cross-linking of three-component biomaterials. The literature review focused on cases where the material was composed of three components and a chemical substance was employed as the cross-linking agent. The most commonly used cross-linking agents identified in the literature include glyoxal, glutaraldehyde, dialdehyde starch, dialdehyde chitosan, and the EDC/NHS mixture. Additionally, the review briefly discusses materials cross-linked with the MES/EDC mixture, caffeic acid, tannic acid, and genipin. Through a critical analysis of current research, this work aims to guide the development of more effective and safer biopolymeric materials tailored for biomedical applications, highlighting potential areas for further investigation and optimization.

## 1. Introduction

Biopolymers, also known as natural polymers, are macromolecular organic compounds widely used across various fields. They find applications in personal care products, the packaging industry, water purification, pharmacy, and the biomedical field [1,2,3,4]. In the context of biomedical applications, natural polymers are employed in tissue engineering, for example, as wound dressings or three-dimensional substrates for cell culture, as well as in drug delivery systems, and as injectable hydrogels or as bioinks for 3D printing [5,6,7,8].

Today, modern biomaterials are often prepared using more than one component. Synthetic and natural polymers can be blended to create biomaterials [8,9]. In recent years, there has been increasing interest in developing new materials based on blends of two or three biopolymers [5,6,7,10]. Such blends can form a new class of materials with enhanced biocompatibility and modified physicochemical properties, including mechanical strength, swelling behavior, degradability, porosity, and thermal stability. Additionally, one of the three components, or an additional component in the blend, can be an inorganic material, such as hydroxyapatite, bioactive glass, zinc oxide, or magnetic particles [11].

There are several review papers on biomaterials made from two- and three-component blends [5,6,9,10,11,12,13]. In 2021, [5] we prepared an article on improving the physicochemical properties of silk fibroin-based materials with the addition of collagen, chitosan, alginate, or hyaluronic acid. We also discussed three-component blends used to obtain biomaterials. The following year, in 2022, we [6] detailed the mixtures of chitosan and silk fibroin for biomedical applications. A review of sustainable biomaterials based on cellulose, chitin, and chitosan composites was prepared by Kostag M. and El Seoud O.A. [12]. They presented fibers, nanofibers, films, membranes, and various shapes as examples of cellulose/chitin/chitosan biocomposites. Two-component collagen-based materials have been reviewed multiple times, as collagen is known to be highly miscible with chitosan, silk fibroin, alginate, cellulose, keratin, elastin, and hyaluronic acid [7,13]. Chitosan composites with proteins (collagen and gelatin), glycosaminoglycans (heparin, chondroitin sulfate, and hyaluronan), genetic materials (DNA and siRNA), polysaccharides (alginate, pectin, carrageenan, starch, cellulose, and dextran), synthetic nondegradable polymers (PVA—polyvinyl alcohol, PCL—poly-ε-caprolactone, PVP—poly(N-vinyl-2-pyrrolidone)), and synthetic degradable polymers (PGA—poly-γ-glutamic acid, PLA—poly(l-lactic acid), and PEG—polyethylene glycol) were described by Hein S. et al. [14] and Seidi F. et al. [15].

There are two main methods for obtaining material-based blends: combining in the molten state (melt mixing) [16] and mixing as aqueous solutions in appropriate solvents [17]. When it comes to obtaining biopolymer-based materials, the second method is safer because the reaction between solids at high temperatures and pressure during melt mixing can be detrimental to proteins, leading to degradation and denaturation [5,8]. It is necessary to explore the interactions between the components and assess their miscibility when producing materials by mixing as aqueous solutions. The most common methods for this analysis are viscometry, differential scanning calorimetry (DSC), and Fourier transform infrared spectroscopy (FTIR) [5,18].

Not only is the mixing of three biopolymers of interest to scientists nowadays but as is their cross-linking. Cross-linking is the process that leads to the creation of new bonds in materials. The main aim of cross-linking is to overcome the limitations of polymeric and biopolymeric materials by increasing molecular weight, generating intermolecular interactions, and interconnecting molecules [19,20,21]. The cross-linking process results in the modification of the physicochemical properties of materials obtained from polymers and biopolymers. It is the most common method used to improve such properties as mechanical strength, stability in water conditions, regularity of pores, thermal stability, and degradation resistance. Three types of cross-linking can be distinguished: enzymatic, physical, and chemical cross-linking. Chemical cross-linking is the most widespread and most commonly used method to modify biomaterial properties. It primarily allows for the formation of very strong bonds between polymer compounds and cross-linking agents [5,22,23]. Additionally, the major advantages of chemical cross-linking are the relative availability of reagents, process efficiency, and predictability. Unfortunately, chemical cross-linking also has its disadvantages: it is more expensive than physical cross-linking. Additionally, after the process, materials need to be washed to remove any residual cross-linker. Furthermore, cell toxicity must be tested because a residual cross-linker could be toxic to cells cultured in these materials [5,19,22]. Despite these disadvantages, scientists most often choose chemical cross-linking. Currently, the state of knowledge about chemical cross-linking is advanced. There are many articles available regarding various cross-linking agents, cross-linker concentrations, and methods for removing residual cross-linker [23,24,25,26,27,28,29,30].

**Figure 1:**
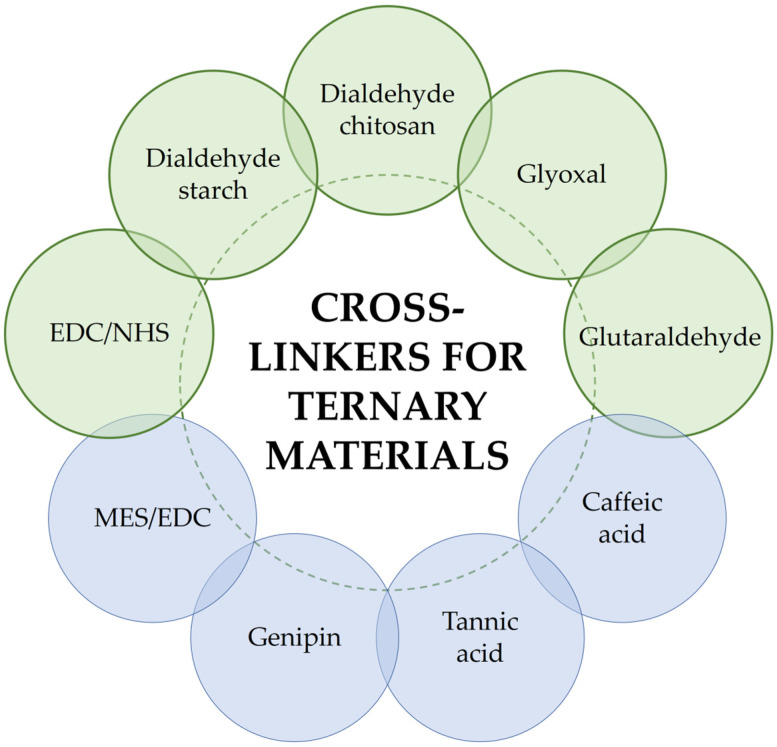
Cross-linking agents for ternary materials.

A variety of different cross-linking agents are used to react chemically with the components of polymer and biopolymer materials, such as glyoxal, glutaraldehyde, various dialdehydes (starch dialdehyde, chitosan dialdehyde, and alginate dialdehyde), squaric acid, or a mixture of 1-Ethyl-3-(3-dimethylaminopropyl) carbodiimide hydrochloride and N-hydroxysuccinimide (EDC/NHS) [5]. In this short review, attention will focus on a few of these agents that are used to modify the physicochemical properties of ternary materials (Figure 1).

## 2. Cross-Linking Agents for Ternary Materials

### 2.1. Glyoxal

**Figure 2:**
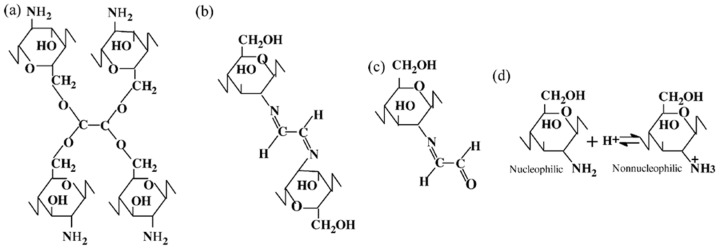
A description of a possible mechanism for glyoxal cross-linking to chitosan: (**a**) glyoxal reacts with hydroxyl groups in chitosan, (**b**) glyoxal reacts with amino groups in chitosan, (**c**) one carbonyl in glyoxal reacts, and (**d**) effect of acid on chitosan nucleophilicity. Adapted with permission from Yang Q. et al. Studies of the cross-linking reaction on chitosan fiber with glyoxal, *Carbohyd. Polym.* **2005**, *59*, 205–210 [36].

Glyoxal (GLY) is a simple organic compound that belongs to the group of dialdehydes. It is the smallest dialdehyde [31,32], and the presence of two adjacent carbonyl groups is responsible for its high reactivity. Glyoxal has high water solubility and can be easily used as a cross-linking agent for proteins and polysaccharides [33,34,35,36,37]. It has been extensively used as a cross-linker for chitosan, cellulose, starch, and chitosan/collagen materials. Kaczmarek-Szczepańska et al. discussed that the glyoxal has a cross-linking ability via acetal formation between the aldehyde groups of glyoxal and the hydroxyl groups of the glucosamine units of chitosan, or Schiff’s base formation between the free amino groups of chitosan or collagen and the aldehyde groups of glyoxal [38]. A possible mechanism of the glyoxal-chitosan reaction has been described in detail by Yang Q. et al. [36], and presented in Figure 2.

Silk fibroin (SF), collagen (Coll), and chitosan (CTS) scaffolds have been prepared by the freeze-drying method, and glyoxal was used to modify the physicochemical properties of these scaffolds [32]. It was found that glyoxal improved the materials’ properties at a low cost because it is one of the cheapest cross-linking agents. The authors used glyoxal as a 5% addition relative to the weight of the polymers in the mixture, and they obtained full cytocompatibility with MG-63 cells for each studied scaffold, but the most adequate environment for cell cultures was the one based on the two-component SF/Coll 50/50 mixture with a 20% chitosan addition. Moreover, the scaffolds were characterized by a porous structure and a high swelling degree [32].

Glyoxal was used as a cross-linking agent to improve the properties of carboxymethyl cellulose/polyvinyl alcohol/polyvinylpyrrolidone blends [39]. The polymers were blended in equal weight (1:1:1 *w*/*w*), and glyoxal was used in three different weight ratios—10, 15, and 20 *w*/*w*%. Hydrogel films were obtained by the solvent-casting method. The results of mechanical testing, swelling behavior, and SEM imaging were compared with the same materials cross-linked with glutaraldehyde. Materials cross-linked with glyoxal showed the highest entanglement, with the highest elongation observed in the hydrogel with 15% addition of glyoxal. Each prepared hydrogel film was characterized by water absorption and homogeneity of the blend components [39]. The authors proposed the materials as candidates for wound dressing applications because of their excellent swelling properties.

### 2.2. Glutaraldehyde

**Figure 3:**
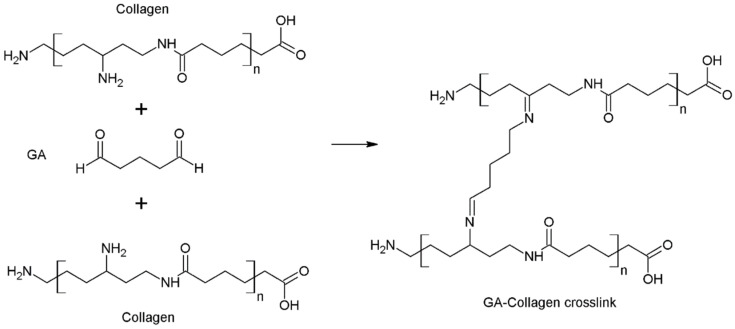
The mechanism of collagen cross-linking reaction using glutaraldehyde. Adapted from Sapuła P. et al. Are Natural Compounds a Promising Alternative to Synthetic Cross-Linking Agents in the Preparation of Hydrogels? *Pharmaceutics* **2023**, *15*, 253 [33].

Glutaraldehyde (GLU) is one of the most commonly employed cross-linking agents due to its superior cross-linking efficiency compared to other aldehydes. It is known for its excellent solubility in both water and organic solvents. The primary reasons for its widespread use in cross-linking reactions are its easy availability, low cost, and high reactivity [33]. It has been used to modify various types of biomaterials, such as films, scaffolds, microparticles, foams, and fibers [40,41]. Although it is an excellent agent for improving the physicochemical properties of materials, some references mention concerns about the toxicity of glutaraldehyde, especially in higher concentrations [42]. There are many reports regarding cross-linking mechanisms, where the aldehyde group from glutaraldehyde reacts with the ε-amino groups, which are derived from the collagen lysine and hydroxylysine residues (Figure 3) [24,33,43].

**Figure 4:**
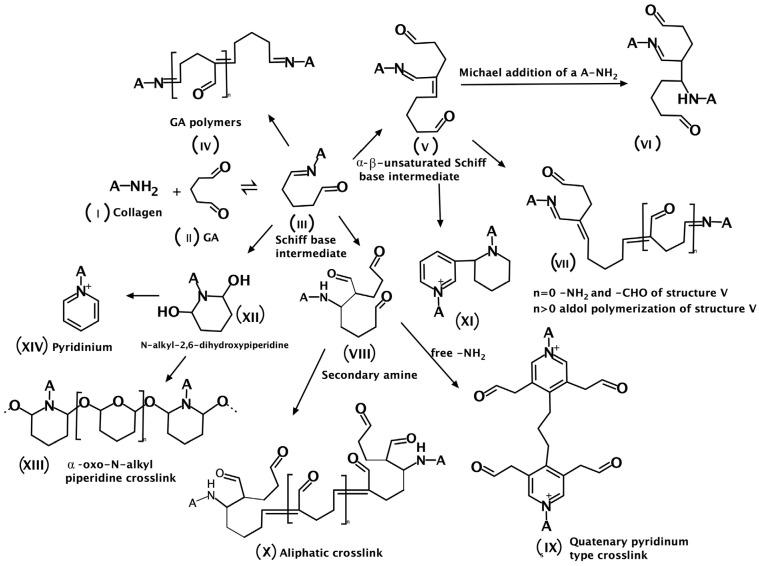
Possible pathways for the cross-linking reaction between glutaraldehyde and collagen (A). Adapted with permission from Adamiak K. and Sionkowska A. Current methods of collagen cross-linking: Review. *Int. J. Biol. Macromol.* **2020**, *161*, 550–560 [24]. Numbers explanation: (I) collagen; (II) glutaraldehyde; (III) the Schiff base intermediate formed from collagen and glutaraldehyde; (IV) glutaraldehyde polymers; (V) α-β unsaturated Schiff base intermediate; (VI) glutaraldehyde polymers the other type of cross-link formed due to Michael addition of collagen amine group to unsaturated Schiff base; (VII) glutaraldehyde polymers with cross-link formed by the reaction of amine groups with free aldehyde groups of (V); (VIII) secondary amine formed as a consequence of a Mannich-type reaction between GA-related enol and protonated Schiff base; (IX) six-membered dihydropyridine—product of the interaction between VIII and other GA molecule and subsequent ring closure; (X) aliphatic cross-links emerging after aldol condensation followed by the reaction with collagen amine groups; (XI) substituted quaternary pyridinium type cross-links obtained by oxygen-induced oxidation of dihydropyridine; (XII) N-alkyl-2,6-dihydroxy piperidine; (XIII) α-oxo-N-alkyl piperidine cross-links formed after condensation of N-alkyl-2,6-dihydroxy piperidine with cyclic monohydrated GA; (XIV)pyridinium.

The Schiff base, which is the main intermediate product of the reaction, constitutes the substrate for the further intermediate responses, which is fully described by Adamiak K. and Sionkowska A. in accordance with Figure 4 [24].

Glutaraldehyde was used to cross-link hydroxyapatite/chitosan-gelatin (Gel) composite scaffolds [44]. The materials were prepared by the phase separation method and had to be treated with sodium borohydride (NaBH_4_) solution to eliminate unreacted glutaraldehyde. A porosity of 90.6% was detected in the scaffolds, and the adhesion, proliferation, and expression of rat calvaria osteoblasts on these highly porous scaffolds were demonstrated. Moreover, after 3 weeks in culture, a good biomineralization effect was observed in the cell/scaffold constructs [44].

Complex films consisting of hyaluronic acid (HA), type I collagen, and chitosan, cross-linked with glutaraldehyde, were obtained, and their physicochemical properties were evaluated [45]. A series of studies were performed, including inverted microscopic observation, atomic force microscopic (AFM) imaging, flow cytometry (FCM) measurement, MTT assay, and MIC assay. The results provided valuable data for the future application of these films as a type of wound dressing material [45].

Electrospun chitosan/polycaprolactone/hyaluronic acid scaffolds for potential wound healing applications were cross-linked with glutaraldehyde. Glutaraldehyde was used to connect two layers of material: a chitosan and polycaprolactone scaffold prepared using electrospinning and hyaluronic acid. The studies showed that using glutaraldehyde is safe, as in vitro studies with Vero cells [an epithelial cell line extracted from the African Green Monkey, *Chlorocebus* sp.] confirmed enhanced growth, proliferation, and migration of Vero cells on the materials based on the chitosan, polycaprolactone, and hyaluronic acid scaffold [46].

As mentioned above, carboxymethyl cellulose/polyvinyl alcohol/polyvinylpyrrolidone-based blends were cross-linked with glutaraldehyde [39]. A similar analysis was conducted to obtain materials like these but cross-linked with glyoxal. The results showed that the mixture of hydrogel films cross-linked with 10 w/w% of glutaraldehyde exhibited the highest elongation at 17.53%. Additionally, as previously noted, the materials demonstrated high water absorption (approximately 190%) [39].

In addition to the examples described above, the glutaraldehyde cross-linking of nanochitosan/polyvinylpyrrolidone/silk fibroin blends is also well-documented [47]. During this experiment, binary nanochitosan/polyvinylpyrrolidone and ternary nanochitosan/polyvinylpyrrolidone/silk fibroin-based blends were cross-linked. Based on FTIR analysis, the corresponding peak shifts and intensity shifts in the spectra of NC/PVP and NC/PVP/SF blends prepared in the presence of the cross-linker glutaraldehyde confirm the formation of the binary and ternary blends with good physicochemical interaction. Additionally, the ternary blend cross-linked with glutaraldehyde was characterized by high thermal stability. Taking into account the results of FTIR, XRD, TGA, and DSC, it can be concluded that glutaraldehyde acted as a superior binding agent [47].

### 2.3. Dialdehyde Starch

**Figure 5:**
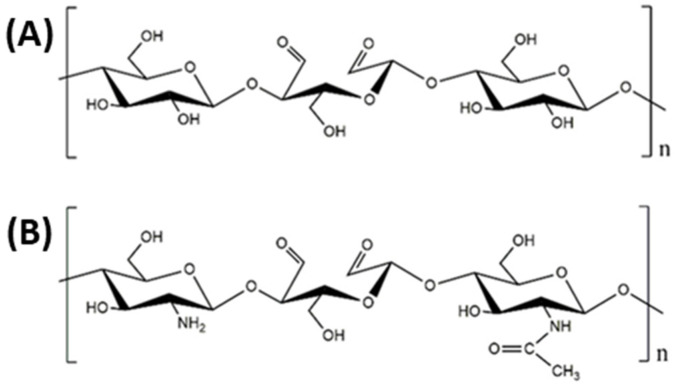
The structure of (**A**) dialdehyde starch and (**B**) dialdehyde chitosan [5].

Dialdehyde starch (DAS, Figure 5A) is a derivative of starch and can be obtained through an oxidation process using sodium periodate or periodic acid as oxidants [48,49]. Dialdehyde starch exhibits excellent chemical, physical, and biochemical properties, such as strong adhesion and alkaline solubility, due to the presence of many active aldehyde groups [50]. It can be used as a cross-linking agent for various proteins (collagen, casein, wheat gluten, and corn zein) [51,52], polysaccharides (chitosan and hyaluronic acid) [53,54], and also as a component of materials used in the biomedical field [54,55,56] and packaging industry [57].

Thin films based on collagen, hyaluronic acid, and chitosan mixtures were prepared and cross-linked with dialdehyde starch [58]. A 5% (*w*/*w*) addition of dialdehyde starch was used to modify the biopolymeric films. The cross-linking led to modifications in the mechanical properties and surface characteristics of the films. The modified films were more resistant to rupture, less elastic, and their surfaces were less rough with higher surface free energy due to the covalent bonds between the carbonyl and amine groups [58]. The Young’s modulus of the Coll/HA 50/50 + 30%CTS cross-linked sample increased by approximately three times (to about 0.62 GPa) relative to the sample not modified by dialdehyde starch (about 0.21 GPa). Moreover, a tensile strength value for Coll/HA 50/50 + 30%CTS with the addition of DAS that was approximately twice as high as that of the pristine sample was also observed. In addition, films modified with dialdehyde starch became more polar, with a higher surface energy value and increased wettability [58].

The same biopolymers—chitosan, collagen, and hyaluronic acid—but in different configurations have been used to obtain scaffolds with the addition of nanohydroxyapatite (nHAp) or as a matrix for calcium phosphate in situ precipitation [59], cross-linked with dialdehyde starch [59,60], or tannic acid [60]. t was observed that scaffolds based on CTS/Coll 50/50 + HA + 50%nHAp had improved mechanical properties when dialdehyde starch was used as the cross-linking agent [59,60]. Moreover, the porosity of scaffolds modified with DAS was higher than that of those in which tannic acid was applied. However, it was also observed that tannic acid enhanced the scaffold’s biological properties (a human osteosarcoma cell line, SaOS-2, was used to study proliferation) [60]. The addition of magnetic particles to the chitosan, collagen, and hyaluronic acid materials cross-linked with dialdehyde starch was also noted [61]. The use of dialdehyde starch to cross-link the materials led to changes in the swelling degree and porosity but had no significant effect on the density of the materials [61].

Scaffolds based on chitosan, collagen, and silk fibroin have been cross-linked with dialdehyde starch, and their physicochemical properties have been evaluated [62,63]. The obtained properties depended on the scaffold composition. They were characterized by high porosity (approximately or more than 90%), a high swelling degree, and interconnected pores (less than 200 µm in size). Materials modified with dialdehyde starch had improved properties, such as enhanced swelling ability, which is important in tissue engineering. All the studied materials (Coll/CTS 50/50 with SF; Coll/SF 50/50 with CTS; and CTS/SF 50/50 with Coll) were cytocompatible with MG-63 cells [62,63].

### 2.4. Dialdehyde Chitosan

**Figure 6:**
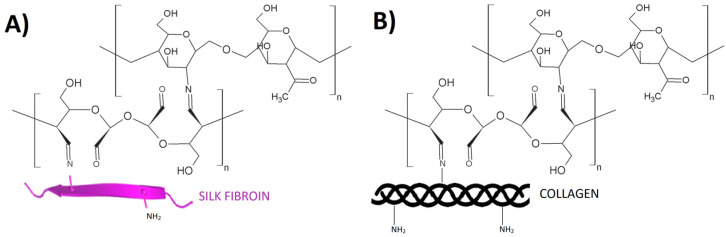
The possible mechanisms of cross-linking between chitosan, a compound with a dialdehyde group (e.g., DAS, DAC), and (**A**) silk fibroin or (**B**) collagen. Adapted from Grabska-Zielińska S. et al. Is Dialdehyde Chitosan a Good Substance to Modify Physicochemical Properties of Biopolymeric Materials? *Int. J. Mol. Sci.* **2021**, *22*, 3391 [5].

Dialdehyde chitosan (DAC, Figure 5B) is a derivative of chitosan. DAC can be synthesized from chitosan by periodate oxidation, which introduces carbonyl groups (C=O) into its structure [64]. The possible mechanisms of cross-linking between polysaccharide (chitosan), a compound with a dialdehyde group (e.g., DAS, DAC), and protein (silk fibroin, collagen) have been shown in Figure 6.

Recent research has shown that dialdehyde chitosan demonstrates significant inhibitory activity against fungal strains, such as *Aspergillus brasiliensis* and *Candida albicans*, compared to native chitosan [64] and exhibits an antimicrobial response against Gram-positive bacteria (*Staphylococcus aureus* and *Bacillus subtilis*) and Gram-negative bacteria (*Escherichia coli* and *Serratia*) [65]. Additionally, DPPH tests confirmed that the antioxidant activity of dialdehyde chitosan is higher compared to that of native chitosan [65].

In addition to studying the influence of dialdehyde chitosan on chitosan films [66] and collagen scaffolds [67,68], it was also used to cross-link the three-component materials for use in tissue engineering [62].

Three types of mixtures based on chitosan, collagen, and silk fibroin (Coll/CTS 50/50 with SF addition; Coll/SF 50/50 with CTS addition; and CTS/SF 50/50 with Coll addition) were cross-linked with dialdehyde chitosan, and their properties were compared with those of the same materials (based on chitosan, collagen, and silk fibroin) cross-linked with dialdehyde starch [62]. The results showed that dialdehyde chitosan impacts properties such as mechanical resistance, pore size, swelling ability, and thermal stability of the materials. In addition to the physicochemical properties studied, a cytocompatibility test with MG-63 cells was conducted, and the results showed that dialdehyde chitosan is not toxic; however, the materials cross-linked with dialdehyde starch provided a better environment for the culture of MG-63 cells [62].

### 2.5. EDC/NHS

**Figure 7:**
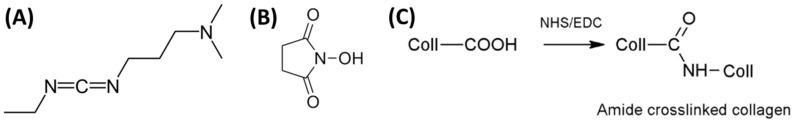
The structure of (**A**) 1-Ethyl-3-(3-dimethylaminopropyl) carbodiimide hydrochloride (EDC), (**B**) N-hydroxysuccinimide (NHS), and (**C**) chemical cross-linking reaction of collagen using EDC/NHS. Adapted from Grabska-Zielińska S. et al. Is Dialdehyde Chitosan a Good Substance to Modify Physicochemical Properties of Biopolymeric Materials? *Int. J. Mol. Sci.* **2021**, *22*, 3391 [5] and Sapuła P. et al. Are Natural Compounds a Promising Alternative to Synthetic Cross-Linking Agents in the Preparation of Hydrogels? *Pharmaceutics* **2023**, *15*, 253 [33].

The mixture of 1-ethyl-3-(3-dimethylaminopropyl) carbodiimide hydrochloride and N-hydroxysuccinimide (structures shown in Figure 7A,B) is a commonly used agent for the cross-linking reaction of polysaccharides and proteins. It is a “zero length” cross-linking agent that chemically activates a molecule, which enables the conjugation of two molecules only through a direct covalent bond without any linker or spacer [24]. During the cross-linking process, the formation of a covalent bond between carboxylic acid groups from aspartic and glutamic acid is induced [24,69]. Figure 7C shows the chemical cross-linking reaction between EDC/NHS with collagen [33].

**Figure 8:**
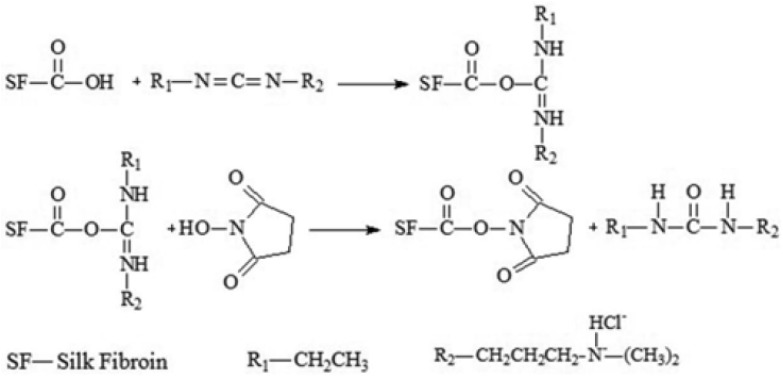
The schematic of the mechanism of crosslinking of silk fibroin with EDC/NHS. Adapted with permission from Liu R. et al. EDC/NHS Crosslinked Electrospun Regenerated Tussah Silk Fibroin Nanofiber Mats. *Fiber. Polym*. **2012**, *13*, 613–617 [71].

The mixture of EDC/NHS was used to modify three-component scaffolds made of collagen, chitosan, and silk fibroin [21]. The EDC/NHS mixture was prepared in 98% ethanol. After that, the scaffolds were immersed in this mixture for 4 h at room temperature, then washed with Na_2_HPO_4_, followed by deionized water, frozen, and lyophilized. The proposed mechanism of silk fibroin and EDC/NHS cross-linking reaction is shown in Figure 8. The resulting samples were characterized by a porous structure, a high swelling degree, moisture content ranging between 9.73 and 18.26 g per 100 g of dry sample, biocompatibility, and cytocompatibility with MG-63 osteoblast-like cells [21].

The same method to obtain EDC/NHS cross-linked scaffolds was used for chitosan/collagen/hyaluronic acid materials [70]. These structures were used as matrices for calcium phosphate in situ precipitation. The prepared three-dimensional scaffolds were characterized by high porosity, biocompatibility, thermal stability, and high mechanical resistance. Moreover, the attachment and proliferation of human osteosarcoma SaOS-2 cells were studied, and the results showed better attachment and proliferation for the modified materials compared to unmodified control samples [70].

A slightly different method of obtaining EDC/NHS cross-linked materials was used in the following studies [72,73,74]. Chitosan, gelatin, and hyaluronic acid were mixed; membranes were obtained by the solvent evaporation method (CTS, Gel, and HA at a mass ratio of 6:3:1) and scaffolds were obtained by the freeze-drying method (CTS, Gel, HA at mass ratios of 6:3:1 and 2:7:1) [72,73,74]. The mixture of EDC and NHS in MES buffer was used to cross-link the membranes and scaffolds. In both cases (membranes and scaffolds), the materials were first pretreated with MES in ethanol solution and then incubated in 30 mM EDC and 8 mM NHS in 50 mM MES buffer. After that, the salt was removed by washing in distilled water. Finally, the cross-linked membranes were dried at room temperature and humidity, whereas the cross-linked scaffolds were lyophilized again to obtain dry structures [72]. To sterilize the obtained scaffolds, ^60^Co γ-irradiation (at 2.5 Mrad) was used [74].

In addition to the above-described EDC/NHS cross-linking methods, one more method can be found [75]. In this experiment, chitosan, chondroitin sulfate (CS), and hyaluronic acid were used as the base materials. Additionally, nanohydroxyapatite (nHAp) was added, and cross-linking with the EDC/NHS mixture was applied. The cross-linking agents EDC and NHS were added to the mixture of chitosan, chondroitin sulfate, and hyaluronic acid according to a mole ratio of EDC:NHS:COOH = 2:1:1 (COOH from the acetic acid, which was the solvent used to dissolve chitosan, chondroitin sulfate, and hyaluronic acid). Based on the resulting mixture, intermediary scaffolds were obtained. First, the mixture was transferred into a 24-well polystyrene plate, cooled at 4 °C, and then frozen at −20 °C, sequentially. Next, the scaffolds were incubated in a 2.5 wt% NaOH ethanol/water solution, repeatedly washed with deionized water, and lyophilized to obtain cross-linked CTS/CS/HA/nHAp scaffolds [75].

### 2.6. Other Agents

**Figure 9:**
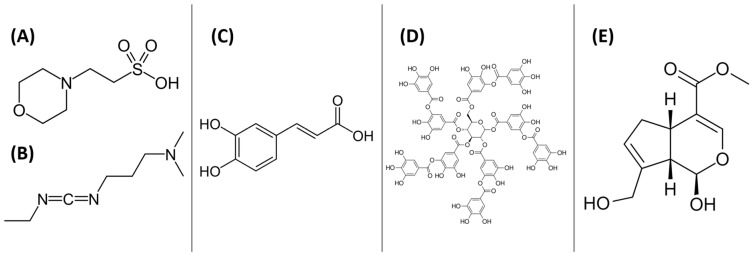
The structures of other agents used to modification of three-component mixtures: (**A**) 2-morpholinoethanesulfonic acid (MES); (**B**) 1-ethyl-3-(3-dimethyl aminopropyl) carbodimide (EDC); (**C**) caffeic acid; (**D**) tannic acid; and (**E**) genipin.

This chapter is devoted to the most commonly used substances for cross-linking biomaterials based on three substrates. However, it is also worth mentioning that there are reports in the literature where other substances have been used to modify the physicochemical properties of three-component mixtures (Figure 9). Below, a few cases are described where ternary materials were cross-linked using a mixture of MES and EDC, caffeic acid, tannic acid, and genipin.

Novel biomimetic tripolymer scaffolds for bone marrow-derived human mesenchymal stem cell-based bone tissue engineering have been prepared using chitosan, collagen type I, and hyaluronic acid [76]. A mixture of 2-morpholinoethanesulfonic acid (MES) and 1-ethyl-3-(3-dimethylaminopropyl) carbodiimide (EDC) (Figure 9A—MES; Figure 9B—EDC) was used as a cross-linking agent to stabilize the scaffold structure. The presence of MES (or phosphate buffer) during the EDC-collagen cross-linking ensures the preferred conditions for performing the reaction, which are acidic or neutral. Based on the results, the authors suggest that these scaffolds hold great promise as cell-delivery vehicles for regenerative therapies and as support systems for enhancing bone regeneration. This is due to their ability to enhance cell proliferation, support cell adhesion, maintain good cell viability, promote cell migration, and exhibit osteogenic potential [76].

**Figure 10:**
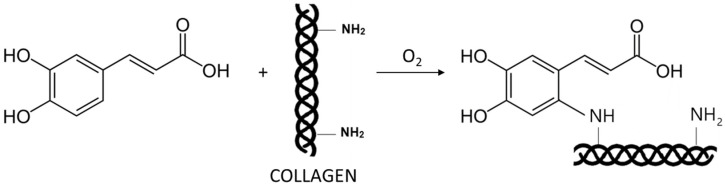
Possible mechanism of the caffeic acid-collagen cross-linking reaction. The scheme is prepared based on Figure 1A in Ji. H et al. Feasibility of caffeic acid as a crosslinking agent in modifying acellular extracellular matrices. *Biochem. Biophys. Res. Commun*. **2023**, *677*, 182–189 [81].

Caffeic acid (Figure 9C) was used as a modifier for chitosan/hydrolyzed collagen/hyaluronic acid-based hydrogels [77]. These materials were prepared using the solvent evaporation method. Caffeic acid has been reported as a natural antioxidant and active therapeutic agent with antimicrobial activity, commonly used in food packaging and biomedical applications [78,79,80]. The possible mechanism of the caffeic acid-collagen cross-linking reaction is shown in Figure 10. The materials modified with caffeic acid exhibited antioxidant activity and a high degree of swelling. They have been developed as wound dressing materials with antioxidant properties.

**Figure 11:**
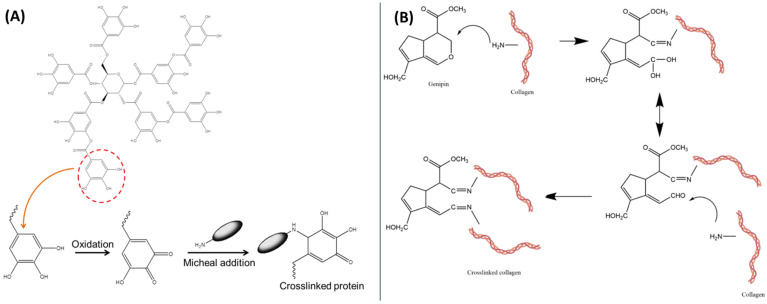
(**A**) Mechanism of crosslinking of proteins/peptides by tannic acid. Adapted from Jayachandran B. et al. Insights on Chemical Crosslinking Strategies for Proteins. *Molecules* **2022**, *27*, 8124 [88]. (**B**) Chemical cross-linking of collagen using genipin. Adapted from Sapuła P. et al. Are Natural Compounds a Promising Alternative to Synthetic Cross-Linking Agents in the Preparation of Hydrogels? *Pharmaceutics* **2023**, *15*, 253 [33].

Tannic acid (Figure 9D), as another phenolic acid, was used as a cross-linking agent [82]. The mechanism of cross-linking of protein/peptides by tannic acid has been showed in Figure 11A. It was added at a 20% weight ratio to materials based on chitosan, collagen, and glycosaminoglycans (GAGs, including hyaluronic acid and chondroitin sulfate) isolated from *Salmo salar* fish skin. The materials were obtained using the freeze-drying method. The results showed that scaffolds based on chitosan, collagen, and glycosaminoglycans cross-linked with tannic acid display properties suitable for tissue engineering applications [82].

Genipin (Figure 9E) is a naturally occurring widely used cross-linking agent with low cytotoxicity and the ability to efficiently cross-link biomacromolecules with primary amino groups, forming intramolecular and intermolecular cross-linking networks [83]. The proposition of chemical cross-linking of collagen using genipin is shown in Figure 11B. It exhibits anti-inflammatory, antifibrotic, and neuroprotective properties [84]. Therefore, it was used to modify collagen/chitosan/hyaluronic acid [85] and collagen/chitosan/hyaluronic acid modified by lysine-based [86] injectable hydrogels for tissue engineering applications [85,86,87]. The results were promising. The authors reported that with the highest concentration of genipin (20 mM), scaffolds with good mechanical properties and prolonged degradation profiles were obtained. The hydrogel structures were compact and durable [85]. Additionally, hyaluronic acid, after modification and cross-linking with genipin, can form covalent bonds with collagen and chitosan. With the appropriate genipin concentration and properly matched content of chitosan, collagen, and modified hyaluronic acid, structurally stable well-defined hydrogels could be obtained [86].

## 3. Critical Comparison of Cross-Linking Agents Commonly Used for Ternary Materials Cross-Linking

Each of the cross-linking agents mentioned above has its own set of disadvantages. Table 1 summarizes the most common limitations associated with the use of chemical cross-linking agents for ternary materials in biomedical applications. Key limitations include cytotoxicity (especially with aldehydes like glutaraldehyde), pH sensitivity, and potential instability in aqueous environments. These factors (Table 1) must be carefully considered when selecting a cross-linking agent, particularly for applications that require biocompatibility and precise control over material properties.

In addition to the specific limitations of each agent, it is also important to consider the general challenges associated with chemical cross-linking methods. Working with agents such as glyoxal, glutaraldehyde, dialdehyde starch, dialdehyde chitosan, and EDC/NHS presents several practical issues. Many of these substances, especially aldehydes like glutaraldehyde, are highly toxic and pose risks through inhalation, skin contact, or ingestion. Therefore, proper organization and use of personal protective equipment are essential when handling these chemicals.

Furthermore, chemical cross-linkers often produce hazardous waste that must be managed and disposed of properly. Aldehydes and carbodiimides can leave behind toxic by-products or unreacted reagents, which can create environmental risks if not discarded correctly. In summary, while chemical cross-linkers are effective at modifying and stabilizing materials, their use carries notable health and environmental risks. Appropriate safety measures, waste management protocols, and careful handling are crucial to minimize these issues and ensure both user safety and environmental sustainability.

## 4. Conclusions and Future Perspectives

To summarize, ternary materials based on polymers with additives are widely used as biomaterials. The properties of ternary materials can be successfully modified to obtain materials with appropriate characteristics. The most popular modification is cross-linking via chemical agents. The summary of the described materials, along with the forms of the biomaterials and the list of studies performed on them, is presented in Table 2.

This review highlights the significant potential of ternary polymer-based materials modified through chemical cross-linking for biomedical applications. The ability to tailor these materials’ physicochemical properties—such as thermal stability, mechanical resistance, and biocompatibility—makes them particularly valuable in fields like tissue engineering, drug delivery, and wound healing. The review underscores the widespread use of cross-linking agents such as glyoxal, glutaraldehyde, and various dialdehydes, which effectively enhance the performance of these materials.

However, despite these advancements, the challenge of developing biomaterials that align with sustainable and eco-friendly practices remains pressing. The field is moving toward the use of natural polymers and cross-linking agents derived from natural sources, which offer promising alternatives to traditional chemical agents. This transition is critical for minimizing environmental impact while maintaining the desired material properties.

Future research should prioritize the development of ternary biomaterials that are both high-performing and environmentally sustainable. This involves focusing on the use of natural polymers and cross-linking agents, particularly those derived from natural sources, such as genipin and modified biopolymers like dialdehyde chitosan or dialdehyde starch. Moreover, exploring new cross-linking agents, such as other dialdehydes (e.g., dialdehyde alginate and dialdehyde cellulose), could yield materials with superior properties, thanks to the enhanced cross-linking ability provided by additional aldehyde groups.

In addition to environmental sustainability, the future of biomaterials research should emphasize the minimalization of chemical agents and solvents, striving to achieve optimal material properties with the least possible environmental footprint. As industry demands for high-performance biomimetic materials continue to grow, the development of three-component systems that closely replicate natural tissue environments will be crucial. These materials should also facilitate the incorporation of cells and drugs, thereby expanding their applicability in advanced biomedical applications.

Ultimately, the continued exploration and innovation in the field of ternary polymer-based materials, with a strong focus on green production practices, will be essential for meeting the evolving challenges in biomedical engineering and for advancing the field in a sustainable and responsible manner.

## Figures and Tables

**Table 1:** The summary of characteristics, applications, and limitations of the chemical agents used for ternary material cross-linking.

Cross-Linking Agent	Characteristics	Applications	Limitations	References
Glyoxal	the smallest existing dialdehyde; the presence of two adjacent carbonyl groups in structure is responsible for its high reactivity	cross-linking of materials based on polysaccharides, peptides, and their mixtures in different forms; stabilization of polymeric structure; improving the physicochemical properties of materials	(1) toxicity: GLY can exhibit cytotoxicity in higher concentrations, which limits its use in biomedical applications (drug delivery, tissue engineering, or medical devices); residual GLY must be thoroughly removed to avoid toxic effects on cells and tissues; (2) pH sensitivity: an acidic environment may suit for GLY to polysaccharides reaction occurrence because the Schiff base reaction takes place in the presence of acid catalysis; (3) concentration: the metabolic activity of hBMSC (human bone marrow stem cells) was not affected by GLY at low concentrations, but there was a relatively clear cut-off point above which cellular activity decreased; (4) exposure time: at a 1 h incubation time, cell metabolic activity was unaffected up, though at increasing exposure time this value was reduced; (5) low efficiency: the steric structure of GLY can be a disadvantage due to a small molecular size and a possibility to forms shorter and potentially less stable cross-links than other aldehydes (glutaraldehyde)	[5,36,89,90]
Glutaraldehyde	the most commonly employed cross-linking agent with high reactivity and efficiency; excellent soluble in water and organic solvents; easy available; low cost	improving the mechanical properties of collagen-based biomaterials; reagent to stabilize a polymer structure against thermal or enzymatic degradation; cross-linking agent for the preparation of heart valves, vascular grafts, elastic cartilages, tendon xerographs, or artificial skin	(1) Coll concentration in material: if the concentration of Coll is too low, the improvement in any important properties (e.g., thermal stability and mechanical strength) is not observed; if the concentration of Coll is too high, the inhomogeneous reaction may occur; (2) toxicity: GLU can exhibit cytotoxicity in higher concentrations, which limits its use in biomedical applications (in vivo medical implants or drug delivery systems); residual GLU must be thoroughly removed to avoid toxic effects on cells and tissues; (3) concentration of cross-linking agent: the limit for GLU concentration is 8% *w*/*v*, which allows for the elimination of the toxic effect; (4) impossibility to cell incorporation: cells cannot be incorporated into materials during fabrication because of the possible cytotoxicity of GLU, and therefore, cells must be seeded onto the exterior of scaffolds post-fabrication; (5) pH sensitivity: the reactivity of GLU is highly dependent on pH; at acidic pH, the aldehyde groups are more reactive, while at neutral or alkaline pH, they are less reactive; it requires careful control of reaction conditions; (6) irritation: GLU is a strong irritant to the skin, eyes, and respiratory system, posing safety concerns during handling and manufacturing processes	[5,24,33,38,89,90,91]
Dialdehyde starch	natural agent; derivative of starch; can be obtained by an oxidation process; exhibits excellent chemical, physical, and biochemical properties due to the presence of active aldehyde groups	cross-linking agent for proteins, polysaccharides, and their mixtures; component of materials used in biomedical field and packaging industry	(1) too low degree of starch oxidation: it results in reduced cross-linking efficiency and may limit its application where high reactivity is required; (2) stability of aldehyde groups: the aldehyde groups in DAS can undergo side reactions, such as hydration or oxidation into carboxylic acids, which can reduce their reactivity over time; this instability can affect the consistency and efficiency of the cross-linking process; (3) limited solubility: DAS is less soluble in water compared to native starch due to the loss of hydroxyl groups during oxidation, which can be problematic in applications where high solubility is required; (4) cytotoxicity: free aldehyde groups can exhibit cytotoxicity, which makes DAS potentially harmful in biomedical applications unless the unreacted aldehyde groups are properly quenched, neutralized or DAS residues will be thoroughly removed	[52,53,54,55]
Dialdehyde chitosan	derivative of chitosan; can be synthesized from chitosan by periodate oxidation; it demonstrates antifungal and antibacterial activity; it exhibits antioxidant activity	cross-linking agent for proteins and polysaccharides and their mixtures; component of materials used in the biomedical field and packaging industry	(1) too low degree of starch oxidation: it results in reduced cross-linking efficiency and may limit its application where high reactivity is required; (2) stability of aldehyde groups: the aldehyde groups in DAC can undergo side reactions, such as hydration or oxidation into carboxylic acids, which can reduce their reactivity over time; this instability can affect the consistency and efficiency of the cross-linking process; (3) poor solubility: DAC has reduced solubility in comparison to native chitosan due to the loss of hydroxyl groups during oxidation, which can be problematic in applications where high solubility is required; (4) cytotoxicity: free aldehyde groups can exhibit cytotoxicity, which makes DAS potentially harmful in biomedical applications unless the unreacted aldehyde groups are properly quenched, neutralized, or DAC residues will be thoroughly removed; (5) synthesis challenges: the oxidation process used to obtain DAC requires the precise control of conditions to achieve the desired level of oxidation; over-oxidation or under-oxidation can lead to inconsistent aldehyde content, affecting the cross-linking performance and the properties of the final material	[62,64,65,66,67,68,92,93]
EDC/NHS	“zero length” cross-linking agent which chemically activates a molecule	cross-linking agent to polysaccharides, proteins and their mixtures; improving physicochemical properties of collagen-based materials	(1) instability of EDC: EDC is water-soluble, hydrolytically unstable, and has a relatively short half-life in water; in water conditions, it tends to rapidly hydrolyze into inactive by-products, which can reduce the reaction efficiency; it needs to be used immediately after preparation; (2) pH sensitivity: the reactivity of EDC/NHS is highly dependent on pH; EDC/NHS has an optimal pH range usually between 4.5 and 7.5; it requires careful control of reaction conditions; (3) cross-linking of Coll with EDC/NHS affects cell adhesion and leads to calcification; (4) cytotoxicity: by-products of EDC (urea derivatives) may be toxic or interfere with biological applications (drug delivery and tissue engineering); residues have to be thoroughly removed	[24,33,88,94,95]

**Table 2:** Summary of three-component materials modified with different cross-linking agents and the studies used to characterized them.

Cross-Linking Agent	Mixture	Form of Biomaterial	Studies	Reference
Glyoxal	Silk fibroin/collagen/chitosan	3D scaffold	FTIR; density; porosity; moisture content; swelling behaviour; SEM; mechanical properties; in vitro cytotoxicity assay with MG-63 cells	[32]
	Carboxymethyl cellulose/polyvinyl alcohol/polyvinylpyrrolidone	Hydrogel films	Mechanical properties; SEM; swelling behaviour	[39]
Glutaraldehyde	Hydroxyapatite/chitosan/gelatin	3D scaffolds	Calvarial osteoblast isolation, seeding, and culture; SEM; immunohistological observation of osteoblasts/scaffold constructs; mineralization study	[44]
	Hyaluronic acid/type I collagen/chitosan	Films	3T3 fibroblast culture; MTT assay; MIC assay; AFM	[45]
	Chitosan/polycaprolactone/hyaluronic acid	3D scaffolds	SEM; FTIR-ATR; mechanical properties; buffer uptake ability; WVTR; wettability; porosity; degradation degree; evaluation of antimicrobial property	[46]
	Nanochitosan/polyvinylpyrollidone/silk fibroin	Films	FTIR; XRD, TGA, DSC	[47]
	Carboxymethyl cellulose/polyvinyl alcohol/polyvinylpyrrolidone	Hydrogel films	Mechanical properties; SEM; swelling behaviour	[39]
Dialdehyde starch	Collagen/hyaluronic acid/chitosan	Films	FTIR; mechanical properties; AFM; contact angle measurements	[58]
	Chitosan/collagen/hyaluronic acid (with nHAp)	3D scaffolds	SEM; porosity; density; liquid uptake; mechanical tests; attachment and proliferation of human osteosarcoma SaOS-2 cells	[60]
	Chitosan/collagen/hyaluronic acid (with calcium phosphate)	3D scaffolds	SEM; EDX; FTIR; porosity; density; mechanical properties; attachment and proliferation of human osteosarcoma SaOS-2 cells	[59]
	Collagen/chitosan/hyaluronic acid (with magnetic particles)	3D scaffolds	ATR-FTIR; density; porosity; SEM; swelling ability; mechanical properties	[61]
	Chitosan/collagen/silk fibroin	3D scaffolds	FTIR-ATR; swelling behaviour; water content; porosity; density; SEM; mechanical properties; thermal properties; cytotoxicity test with MG-63 cells	[62]
	Collagen/silk fibroin/chitosan	3D scaffolds	FTIR-ATR; swelling behaviour; water content; porosity; density; SEM; mechanical properties; thermal properties; cytotoxicity test with MG-63 cells	[62]
	Silk fibroin/chitosan/collagen	3D scaffolds	FTIR-ATR; swelling behaviour; water content; porosity; density; SEM; mechanical properties; thermal properties; cytotoxicity test with MG-63 cells	[62]
Dialdehyde chitosan	Chitosan/collagen/silk fibroin	3D scaffolds	FTIR-ATR; swelling behaviour; water content; porosity; density; SEM; mechanical properties; thermal properties; cytotoxicity test with MG-63 cells	[62]
	Collagen/silk fibroin/chitosan	3D scaffolds	FTIR-ATR; swelling behaviour; water content; porosity; density; SEM; mechanical properties; thermal properties; cytotoxicity test with MG-63 cells	[62]
	Silk fibroin/chitosan/collagen	3D scaffolds	FTIR-ATR; swelling behaviour; water content; porosity; density; SEM; mechanical properties; thermal properties; cytotoxicity test with MG-63 cells	[62]
EDC/NHS	Collagen/chitosan/silk fibroin	3D scaffolds	FTIR; porosity; density; swelling behaviour; moisture content; liquid uptake; SEM; mechanical properties; in vitro cytocompatibility assay with MG-63 cells	[21]
	Chitosan/collagen/hyaluronic acid	3D scaffolds	FTIR; SEM; porosity; density; mechanical tests; EDX; adhesion and proliferation of human osteosarcoma SaOS-2 cells studies	[59]
	Chitosan/gelatin/hyaluronic acid	Membranes	SEM; XPS; PBS solution adsorption; mechanical testing; in vitro degradation; cytocompatibility with human dermal fibroblasts	[72]
	Chitosan/gelatin/hyaluronic acid	3D scaffolds	SEM; XPS; PBS solution adsorption; mechanical testing; in vitro degradation; cytocompatibility with human dermal fibroblasts	[72]
	Chitosan/gelatin/hyaluronic acid	3D scaffolds	SEM; water uptake ability; mechanical properties; FTIR; in vitro biodegradation; construction of artificial dermis in vitro	[73]
	Chitosan/gelatin/hyaluronic acid	3D scaffold	Water uptake and retention abilities; SEM; culture of fibroblast in scaffolds; attachment and proliferation assay; co-culture of keratinocytes and fibroblasts in scaffolds	[74]
MES and EDC	Chitosan/collagen type I/hyaluronic acid	3D scaffolds	SEM; swelling ratio; hMSCs culture on the scaffolds; calcein-propidium iodide live-dead assayed; osteoblast differentiation studies; alkaline phosphatase assay; immunofluorescent staining for fibronectin and osteocalcin	[76]
Caffeic acid	Chitosan/hydrolyzed collagen/hyaluronic acid	Hydrogels	X-ray diffraction; DSC; thermogravimetric analysis; SEM; swelling behaviour; release of caffeic acid; antioxidant activity; total phenolic content; in vitro degradation behaviour; water solubility; WVTR	[77]
Tannic acid	Chitosan/collagen/glycosaminoglycans (GAGs, including HA and CS)	3D scaffold	FTIR-ATR; SEM; porosity; density; mechanical tests; attachment and proliferation of human osteosarcoma SaOS-2 cells	[82]
Genipin	Collagen/chitosan/hyaluronic acid	Hydrogels	Swelling properties; wettability measurements; SEM; mechanical testing; enzymatic degradation studies; Alamar Blue cell viability test; cell morphology analysis	[85]
	Chitosan/collagen/modified hyaluronic acid	Hydrogels	Swelling ability; contact angle measurements; mechanical properties; enzymatic degradation evaluation; SEM; density; porosity; in vitro osteoblasts culture; antibacterial activity assessment	[86]

## Data Availability

No new data were created or analyzed in this study. Data sharing is not applicable to this article.
